# Brain network alterations in individuals with and without mild cognitive impairment: parallel independent component analysis of AV1451 and AV45 positron emission tomography

**DOI:** 10.1186/s12888-019-2149-9

**Published:** 2019-06-03

**Authors:** Yuan Li, Zhijun Yao, Yue Yu, Ying Zou, Yu Fu, Bin Hu, for the Alzheimer’s Disease Neuroimaging Initiative

**Affiliations:** 1 School of Information Science and Engineering, Shandong Normal University, Jinan, Shandong Province 250358 People’s Republic of China; 2 School of Information Science and Engineering, Lanzhou University, Lanzhou, Gansu Province China

**Keywords:** Parallel independent component analysis, multivariate data analysis, Amyloid imaging, Tau imaging, Networks

## Abstract

**Background:**

Amyloid β (Aβ) and tau proteins are considered as critical factors that affect Alzheimer’s disease (AD) and mild cognitive impairment (MCI). Although many studies have conducted on these two proteins, little study has investigated the relationship between their spatial distributions. This study aims to explore the associations of spatial patterns between Aβ deposition and tau deposition in patients with MCI and normal control (NC).

**Methods:**

We used multimodality positron emission tomography (PET) data from a clinically heterogeneous population of patients with MCI and NC. All data were obtained from the Alzheimer’s Disease Neuroimaging Initiative (ADNI) database containing information of 65 patients with MCI and 75 NC who both had undergone AV45 (Aβ) and AV1451 (tau) PET. To assess the spatial distribution of Aβ and tau deposition, we employed parallel independent component analysis (pICA), which enabled the joint analysis of multimodal imaging data. pICA was conducted to identify the significant difference and correlation relationship of brain networks between Aβ PET and tau PET in MCI and NC groups.

**Results:**

Our results revealed the strongly correlated network between Aβ PET and tau PET were colocalized with the default-mode network (DMN). Simultaneously, in comparison of the spatial distribution between Aβ PET and tau PET, it was found that the significant differences between MCI and NC were mainly distributed in DMN, cognitive control network and visual networks. The altered brain networks obtained from pICA analysis are consistent with the abnormalities of brain network in MCI patients.

**Conclusions:**

Findings suggested the abnormal spatial distribution regions of tau PET were correlated with the abnormal spatial distribution regions of Aβ PET, and both of which were located in DMN network. This study revealed that combining pICA with multimodal imaging data is an effective approach for distinguishing MCI patients from NC group.

**Electronic supplementary material:**

The online version of this article (10.1186/s12888-019-2149-9) contains supplementary material, which is available to authorized users.

## Background

Amyloid β (Aβ) and tau proteins have been recognized as two important factors that cause Alzheimer’s disease (AD) and Mild cognitive impairment (MCI) [1, 2]. Many studies have focused on these two proteins separately [3, 4], and Brier MR et al. [5] have calculated the correlation between the deposition of the two proteins on the voxel-wise based on neuroimages. However, little imaging study has investigated the correlated brain networks of these two proteins thus far.

Several brain image studies have been carried out with different technologies [6, 7]. Multivariate statistical paradigms (such as principal component analysis (PCA) or independent component analysis (ICA)) assess distributed alterations and their interrelationships in multiple neuroimaging data. ICA is a data-driven analysis method to study brain networks conducted by neuroimaging. It was widely used in functional magnetic resonance imaging (fMRI) [8, 9], magnetoencephalography [10], electroencephalography [11], structural MRI [12], and PET imaging [13]. As a variation of ICA, parallel ICA (pICA) could estimate independent components in multimodal data [14]. A prior study reported that the multivariate techniques could be sensitive for early diagnosis of AD [15]. pICA was used to identify the mechanism of Aβ deposition that leads to neurodegeneration and cognitive decline in MCI and AD patients [16, 17]. Moreover, Fu L et al. [18] conducted on the spatial correlation network of Aβ protein and fluorodeoxyglucose (FDG).

Study has found the regions of interest (ROI) were correlated with the scale scores in MCI patients [19], while other paper examined whether ROI regions contribute to distinguish patients from normal people [20]. Tapan Gandhi et al. [21] utilized K-fold cross-validation method to validated the wavelet coefficients of EEG data and pointed out that K-fold cross-validation was a rigorous method.

The goal of our research includes two aspects. Firstly, we use pICA method to explore the significant difference and correlation of spatial distribution between AV1451 (tau PET) and AV45 (Aβ PET). Secondly, we combine pICA with multimodal imaging data to distinguish MCI patients from NC group.

## Materials and methods

### Subjects

Tau PET and Aβ PET images were downloaded from the Alzheimer’s Disease Neuroimaging Initiative(ADNI) website (http://adni.loni.usc.edu/), belonging to ADNI-3 phase [22]. The unified preprocessing description of the collected PET data in ADNI database was added in Additional file 1. A total of 140 individuals (65 patients with MCI and 75 NCs) who both had tau and Aβ PET images were included for pICA analysis.

We also recorded scores for the Mini–Mental State Examination (MMSE) [23], and Clinical Dementia Rating (CDR) [24] from the ADNI database as well as the CSF-Aβ value and CSF-Tau value.

### Data acquisition and preprocessing

The acquisition parameters for all scanners have been described in the Additional file 1. PET images were coregistered, averaged, normalized (standardized image and voxel size), and smoothed to produce a uniform resolution (8 mm full-width at half-maximum). PET scans require dynamic 30-min six-frame (5-min each) acquisition beginning 30 min after the injection of 18F-labeled AV1451 and 18F-labeled AV45. We normalized all images spatially according to the PET Montreal Neurological Institute (MNI) brain space template; subsequently, we scaled and averaged the same images using Statistical Parametric Mapping 12 (SPM12: https://www.fil.ion.ucl.ac.uk/spm/software/spm12/) by MATLAB 2014a on the Centos 6.5 operating system. The images adopted were acquired using Siemens, GE, and Philips PET scanners in a resting state. The spatial normalization included a 12-parameter affine transformation; this process was followed by a nonlinear iterative spatial transformation using SPM12.

### pICA

More details about pICA were introduced in [25]. Utilizing multimodal imaging data, pICA identifies the independent components of each image modality. It also estimates the correlation between these components, as well as different image modality. Using Akaike Information Criterion (AIC) and Minimum Description Length Criterion, the number of independent components of each mode were identified [20]. In order to balance the fitting accuracy and complexity of the independent component model, we chose the lowest independent component set of AIC values. In each modality, the contribution of each independent component to the variance across all subjects is expressed by the loading parameters performed in pICA analysis. Making all the components more intuitive, we set the z-score to be |z| > 2.5. The number of independent estimated components is eight [18]. In this study, independent components of tau PET and Aβ PET were identified by pICA method, furthermore the most significantly different regions in tau PET and Aβ PET among patients with MCI were found. For tau PET spatial distribution, the voxel-wise two-sample t-test was used to found the significantly different components between MCI patients and NC. The ROI features for later analysis were identified by components with significant differences. Aβ PET carried on the same process.

Moreover, the Pearson’s correlation coefficients for all pairs of tau PET and Aβ PET independent components were calculated and the variations of age, sex, and all the statistical values were assessed. Significant relationships between Aβ and tau accumulation were measured by Pearson’s correlation coefficients, and these coefficients should be corrected for multiple comparisons. The false discovery rate (FDR) was performed (*p* < 0.05) on the results. Classification analysis separately assessed the contribution of each component to the classification of MCI and NC.

### K-fold cross-validation

The process of cross-validation is to divide the data into subsets, and then select one subset for calculation, while other remaining subsets are used to verify the accuracy of the previous analysis. It considers the initial subset as the training set and the other subsets as the test set [26]*.*

K-fold cross-validation is a common data analysis method. The advantage of K-fold cross-validation is that all samples are used for training and testing, and each sub-sample is treated as a test data only once. Based on our sample size, we set the K value as 5. Therefore, all data were randomly divided into five groups. Four groups were combined in the pICA, and the last group was used to detect the validity of ROI features extracted from the pICA results. The above process was repeated for five times. A single estimation was obtained from the averaged 5 times calculation values [27].

### Statistical analysis

A two-sample *t* test was conducted to identify any significant differences in age or MMSE, CSF-tau and CSF-Aβ. The Mann-Whitney test was conducted to identify any significant difference in CDR score. A chi-square test was performed to identify significant differences with respect to sex or between patients who were APOE4 carriers and noncarriers.

## Results

### Patient characteristics

The characteristics of all 140 subjects are listed in Table 1. No significant difference was observed in sex, age, or APOE4 between the MCI and NC groups. Cognitive performance, estimated from CDR and MMSE results, was significantly worse in MCI group than in NC group.

**Table 1:** Demographic data of all subjects

	MCI	NC	*p*
N (total *N* = 80)	65	75	–
Age	73.27 ± 5.75	76.27 ± 6.22	0.3721^b^
Genger (male:female)	71:69	65:75	0.423^a^
APOE4 (carriers:noncarriers)	32:33	33:42	0.348^b^
MMSE	25.7 ± 2.3	27.9 ± 1.7	< 0.001^a^
CDR	0.5	–	< 0.001^c^
CSF-Tau	251.3 ± 104.9	254.6 ± 128.2	0.89^a^
CSF-Aβ	1036 ± 358	875 ± 321	0.30^a^

Data are presented as a mean ± standard deviations. *p* was obtained using ^a^the two-sample t test, ^b^the chi-square test and ^c^ the Mann-Whitney test

### Individual tau PET and Aβ PET components

**Fig. 1:**
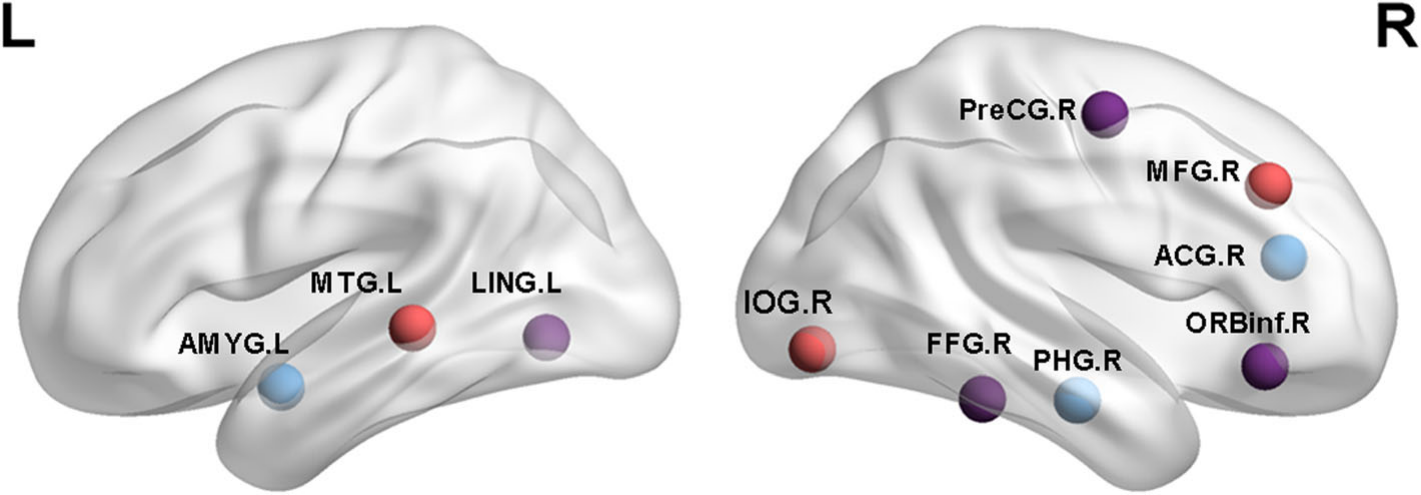
Purple nodes: significant differences between the MCI and NC groups in AV1451 were observed in right fusiform gyrus (FFG.R), left lingual gyrus (LING.L), right inferior frontal gyrus(orbital part) (ORBinf.R), and right precentral gyrus (PreCG.R). Red nodes: significant differences were noted in the left middle temporal gyrus (MTG.L), right inferior occipital gyrus (IOG.R), and right middle frontal gyrus (MFG.R). Blue nodes: significant differences were observed in the right parahippocampal (PHG.R), left amygdala (AMYG.L), right anterior cingulate and paracingulate gyri (ACG.R)

**Fig. 2:**
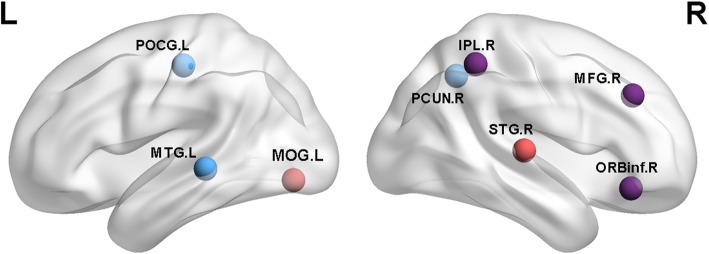
Purple nodes: significant differences between the MCI and NC groups in AV45 were observed in the right middle frontal gyrus (MFG.R), right inferior frontal gyrus(opercular part) (ORBinf.R), right inferior parietal but supramarginal and angula gyri (IPL.R). Red nodes: significant differences were noted in the right middle occipital gyrus (MOG.R), right superior temporal gyrus (STG.R). Blue nodes: significant differences were observed in the left middle temporal gyrus (MTG.L), left postcentral gyrus (POCG.L), and right precuneus gyrus (PCUN.R)

Each kind of data was found three components with significant differences between MCI and NC group. They were discovered to frequently occur (see Table 2 (tau), Table 3 (Aβ), Fig. 1 (tau), and Fig. 2 (Aβ)). We recorded the maximum |z| and *P* values in each respective region. We detected the networks with significant differences in tau PET group as follows: visual network (VN) including right fusiform gyrus; left lingual gyrus; left middle temporal gyrus; right inferior occipital gyrus. The cognitive control network (CCN) including right inferior frontal gyrus (opercular part); right precentral gyrus; right middle frontal gyrus; right parahippocampal gyrus. The default-mode network (DMN) including left amygdala; right anterior cingulate and paracingulate gyri. We detected significant differences in the following networks in Aβ PET group: The VN including right middle occipital gyrus. The CCN including the right middle frontal gyrus; right inferior frontal gyrus (opercular part); right inferior parietal but supramarginal and angular gyri; left postcentral gyrus; right superior temporal gyrus. The DMN including left middle temporal gyrus and right precuneus gyrus.

**Table 2:** Components with significant differences in tau

Brain regions	|z|	Networks	*P* value (components)	X	Y	Z
Fusiform_R	3.523	visual	0.0362	38	−11	−30
Lingual_L	3.176	visual	0.0362	−12	−93	−14
Frontal_Inf_Orb_R	3.298	cognitive	0.0362	48	33	−11
Precentral_R	3.517	cognitive	0.0362	58	12	43
Temporal_Mid_L	3.81	visual	0.0210	−43	−57	−7
Occipital_Inf_R	3.047	visual	0.0210	33	−57	−9
Frontal_Mid_R	3.425	cognitive	0.0210	30	40	28
ParaHippocampal_R	3.628	cognitive	0.0013	24	−5	22
Amygdala_L	3.624	subcortical	0.0013	−23	−6	−21
Cingulum_Ant_R	4.117	DMN	0.0013	−2	−18	22

Abbreviation: *Frontal_Mid_R* Right middle frontal gyrus, *Frontal_Inf_Orb_R* Right Inferior frontal gyrus(orbital part), *Parietal_Inf_R* Right Inferior parietal, but supramarginal and angular gyri, *Occipital_Mid_R* Right Middle occipital gyrus, *Temporal_Sup_R* Right Superior temporal gyrus, *Temporal_Mid_L* Left Middle temporal gyrus, *Postcentral_L* Left Postcentral gyrus, *Precuneus_R* Right Precuneus

**Table 3:** Components with significant differences in Aβ

Brain regions	|z|	Networks	*P* value (component)	X	Y	Z
Frontal_Mid_R	2.81	cognitive	0.0358	24	35	33
Frontal_Inf_Orb_R	2.97	cognitive	0.0358	38	15	31
Parietal_Inf_R	3.027	cognitive	0.0358	36	−41	42
Occipital_Mid_R	4.461	Visual	0.0117	20	−86	19
Temporal_Sup_R	3.312	cognitive	0.0117	53	−38	19
Temporal_Mid_L	2.91	DMN	0.0095	55	−20	−8
Postcentral_L	3.72	cognitive	0.0095	−27	−42	62
Precuneus_R	3.327	DMN	0.0095	0	−53	25

Abbreviation: *Frontal_Mid_R* Right middle frontal gyrus, *Frontal_Inf_Orb_R* Right Inferior frontal gyrus(orbital part), *Parietal_Inf_R* Right Inferior parietal but supramarginal and angular gyri, *Occipital_Mid_R,* Right middle occipital gyrus, *Temporal_Sup_R* Right superior temporal gyrus, *Temporal_Mid_L* Left Middle temporal gyrus, *Postcentral_L* Left Postcentral gyrus, *Precuneus_R* Right Precuneus

### Correlated tau PET and Aβ PET components

**Fig. 3:**
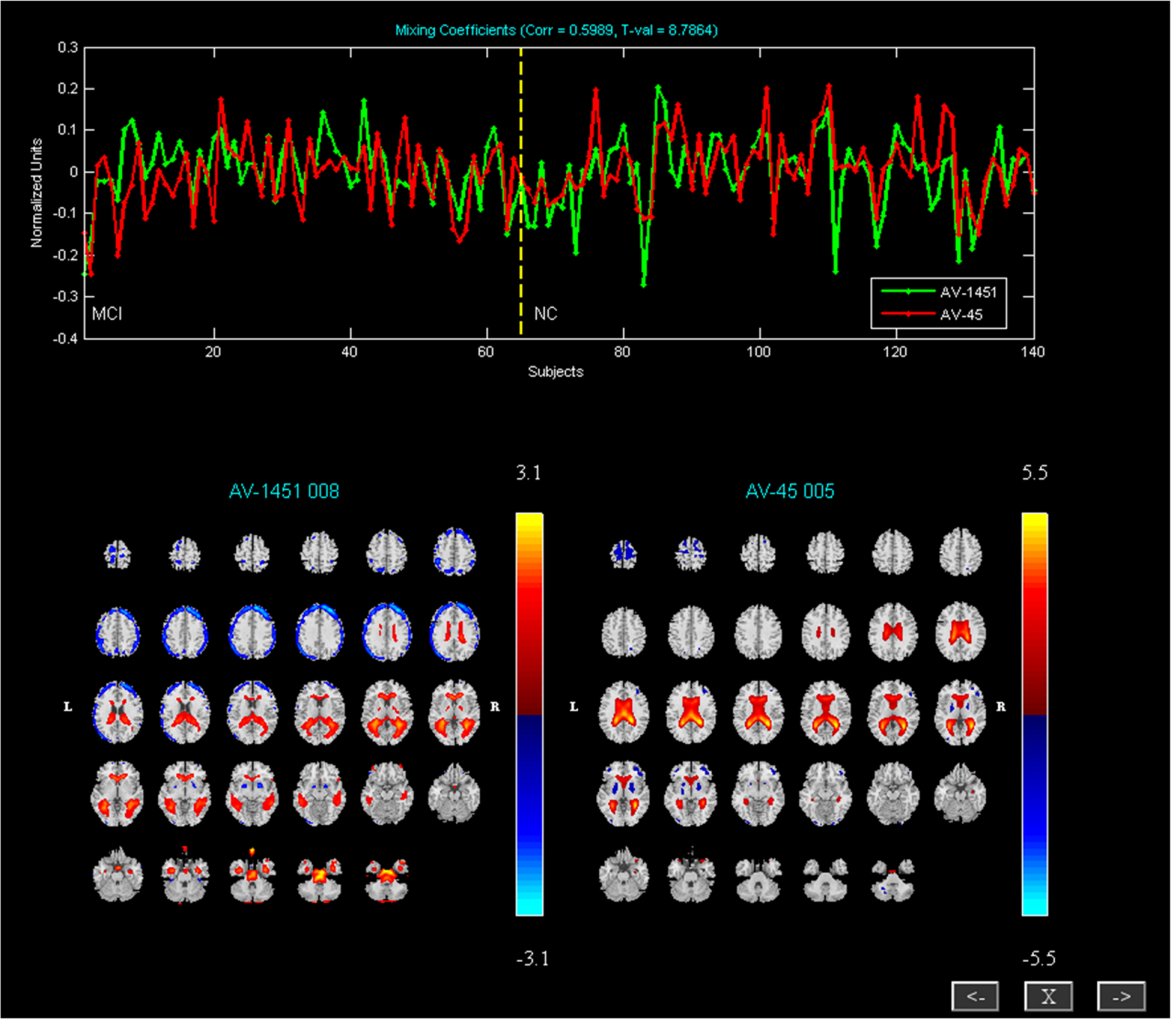
Correlated components of AV1451 and AV45. The top presents loading parameters with a significant correlation in all participants with AV1451 (green) and AV45 (red). Correlated components of AV1451 (left) and AV45 (right), including the medial frontal gyrus; anterior cingulate cortex; posterior cingulate cortex; precuneus; superior temporal gyrus

We also found the correlated networks. One pair of components with the highest correlation (R = 0.5989) was identified between the tau PET and Aβ PET. They were largely colocalized with the DMN. These components mainly contained bilateral precuneus, bilateral angular gyrus, left anterior cingulate cortex, left superior frontal gyrus, left middle temporal gyrus, left middle frontal gyrus, left inferior frontal gyrus (Fig. 3).

### Feature test

**Fig. 4:**
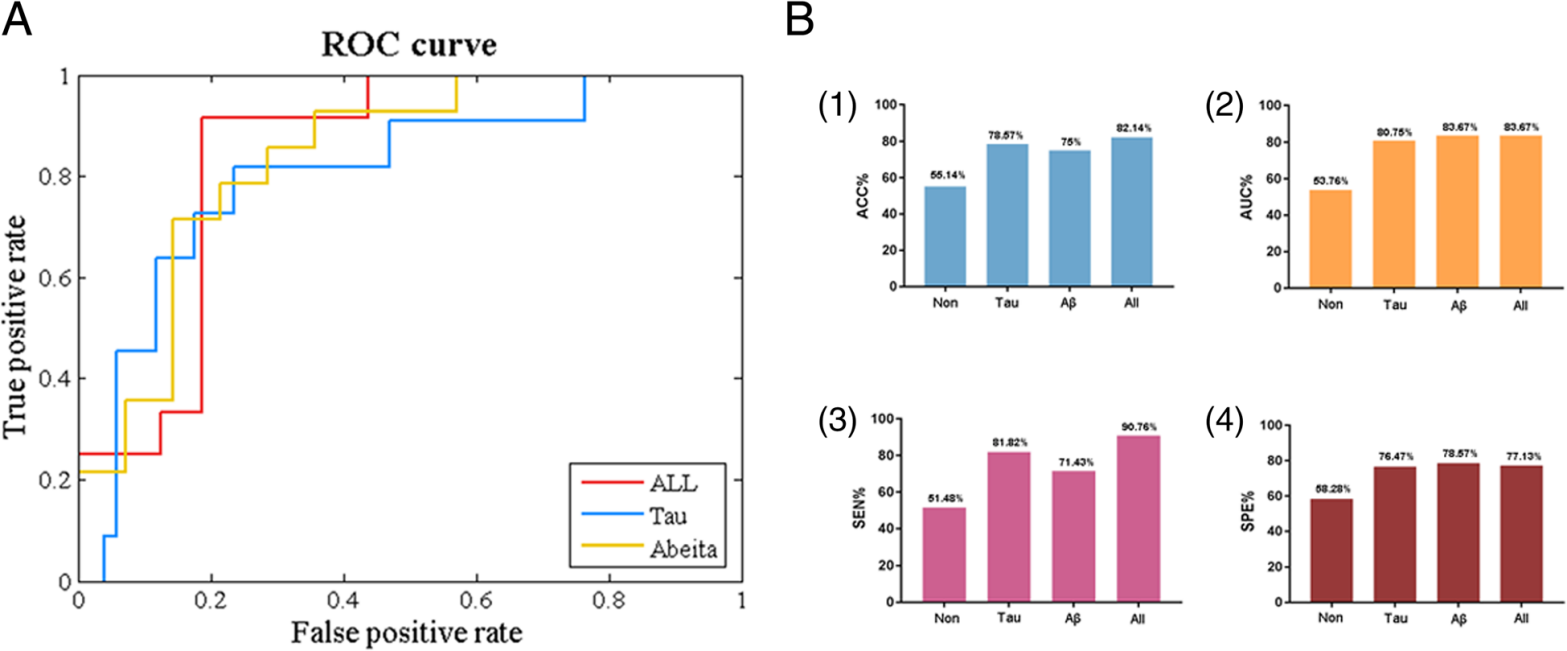
**a** Receiver operating characteristic (ROC) curves of different types of features. Different colors were used to represent the ROC curves of all four types of features. **b** Comparison of the classification accuracy (1); area under the curve (2); sensitivity (3); specificity (4)

Using the method of five-fold cross validation, we detected the regions with significant differences extracted by pICA analysis in distinguishing MCI from NC group. Figure 4 and Table 4presented the contribution of these differential components in the two proteins to classification. The final improvements in the ACCs of all features and the AUCs were respectively 78.57 and 80.75% for tau protein, 75 and 83.67% for Aβ protein, and 82.14 and 84.38% after the fusion of the two proteins. In the original data, the accuracy rate was only slightly more than 50%. The accuracy has been improved and the area under the curve (AUC) has been increased, which suggested features obtained by pICA analysis were effective. The mean values of ACC, AUC, Sensitivity (SEN), and Specificity (SPE) were from five experiments (Table 4).

**Table 4:** Classification results

	ACC	AUC	SEN	SPE
Non	55.14	53.76	51.48	58.28
Tau	78.57	80.75	81.82	76.47
Aβ	75	83.67	71.43	78.57
All	82.14	83.67	90.76	77.13

## Discussion

### Group comparison of tau PET and Aβ PET in brain networks

MCI patients exhibited that components with significant differences for tau and Aβ protein were mainly in DMN, VN, CCN and subcortical networks (SN). These networks are parts of the intrinsic connected networks (ICNs) of human brain. ICNs exhibit a consistent distribution of activity during rest and tasks, which are associated with specific neurocognitive functions [28]. DMN has been considered as a critical role in supporting internal mentation and monitoring external environment [29]. Evidence suggested that Aβ deposition was most likely to occur in DMN, probably due to high synaptic activity [30]. Tau deposition is primarily targeted at high-level cognitive networks such as DMN [31]. Altered connections within DMN in AD and MCI have also been reported in the study [32]. Greicius MD et al. [33] demonstrated that there were close links between DMN and episodic memory processing. Visual function is a major complex sensory domain affected by mental diseases [34]. The abnormalities in VN are associated with the aberrant processing of visual information and visual hallucinations [35]. A prior study indicated that the impairment of ventral visual function, including wrong recognition of an object, face and color, were also well noted in MCI patients [36]. Therefore, our results might discover the abnormalities of the visual network in MCI patients may be affected by the presence of tau and Aβ proteins. Several studies suggested that emotion regulation involved increased activity in cortical regions was associated with CCN [37]. The impaired response of CCN to verbal memory is partly responsible for the decline of memory ability in AD/MCI patients [38]. In addition, the Aβ accumulation is a major trait of the pathogenesis of dementia. Recent studies has observed that there was considerable spatial overlap of Aβ accumulating regions with other ICNs, such as CCN [39]. Hansson O et al. [31] indicated that the regional deposition of hyperphosphorylated tau aggregates in AD generally affected CCN. It has been known that amygdala belonging to SN has a central role in emotional learning and memory [40]. Further, recent morphological analysis suggested there was substantial atrophy within the amygdala in AD/MCI [41]. According to aforementioned findings, it can preliminarily speculate that tau and Aβ proteins have an effect on the brain network of MCI patients. Impairments in brain network may lead to mental dysfunction in MCI patients.

### Strongly correlated tau PET and Aβ PET networks

This study also elucidated spatially disparate relationships between the patterns of tau and Aβ deposition across a heterogenous MCI population. We discovered that a significant correlated pair of components between tau PET and Aβ PET were identified using pICA. Tau protein levels in the bilateral precuneus, right angular gyrus, left anterior cingulate cortex, left angular gyrus, left superior frontal gyrus, and left middle temporal gyrus were strongly correlated to Aβ protein levels in the precuneus, bilateral angular gyrus, left middle frontal gyrus, and left inferior frontal gyrus. They are largely colocalized with the DMN [42]. It was well-documented that the alterations in the brain structure, function, and cognition in MCI patients were related with alterations in brain networks [43]. Utilizing resting state functional connectivity MRI (rs-fMRI), networks correlations have been detected in patients with MCI. These networks mainly were involved in DMN and other networks [44]. Although MCI is related with widespread disruption of network connections, DMN is usually most affected. As a sensory-visceromotor link related to social behavior, emotional control and motivation drive, DMN played many potential roles and had a great relationship with personality composition [45]. Therefore, in MCI, the brain regions associated with DMN were damaged, resulting in phenomenon of metabolic reduction and amyloid abnormalities [46]. In the context of current models of the AD pathophysiological cascade [47], our findings might indicate that Aβ diffusion was similar to that of tau and that the spatial distribution of Aβ and tau may be strongly correlated. Several studies pointed out that Aβ aggregation may be driven by the total flow of neuronal activity, while tau aggregation may be driven by transneuronal spread, generating patterns of neurodegeneration that coincide with specific functional networks and ultimately lead to specific clinical phenotypes [48], which were similar to results in present results.

### Role of multivariate analysis

Multivariate techniques are widely used in neuroimaging data analysis. Multivariate methods mainly focus on the level of brain regions to analyze the correlation and covariance of brain regions. The advantage of these methods is that different modal neuroimaging data can be combined to represent pathophysiology of a disease comprehensively [49]. Unlike the univariate method, multivariate analysis has obvious advantages in studying the mechanism of interregional brain cooperation [50]. Results from multivariate analysis can be seen as a feature of neural network, which is an important perspective to study the brain damage induced by mental illness [51, 52]. In order to guarantee the statistical results more accurate, conservative corrections for voxel wise multiple comparisons were added in the multivariate methods. All in all, the use of multivariate methods will help to obtain more discriminatory features in diagnostic classification. In this study, the combined analysis of tau PET and Aβ PET performed better than that of tau PET and Aβ PET alone in distinguishing MCI patients from NC group.

## Limitations

This study has several limitations. Firstly, pICA assumes that measurements in each image voxel are independent and that the overall noise is uniformly distributed, and these assumptions may not be entirely accurate for PET data. Therefore, future research should focus more on the diversity of data. Secondly, the lack of AD subjects in our research is another limitation due to the incomplete data of ADNI database. After collecting enough AD data in the next step, we will plan to conduct a comparison and combination study of AD and MCI data separately.

## Conclusions

In the present study, we explored the tau PET and Aβ PET spatial distribution pattern in MCI patients and NCs. The pICA results revealed that the abnormal pattern detected by tau PET was in agreement with the abnormal pattern detected by Aβ PET, both of which shared the location of the DMN. Moreover, these regions were helpful for distinguishing patients with MCI from those in the NC group. These results indicated that tau PET and Aβ PET are reliable biomarkers of neurological function and might be helpful for diagnosis.

## Additional file

Additional file 1: Acquisition parameters of PET data. We have revised in the manuscript. (DOCX 13 kb)

## Abbreviations

ACC: Accuracy
AD: Alzheimer’s disease
ADNI: Alzheimer’s Disease Neuroimaging Initiative
APOE4: Apolipoprotein E ε4 allele
AUC: Area under the curve
Aβ PET: AV45
Aβ: Amyloid β
CCN: Cognitive control network
CDR: Clinical Dementia Rating
DMN: Default-mode network
FDG: Fluorodeoxyglucose
fMRI: Functional magnetic resonance imaging
ICNs: Intrinsic connected networks
MCI: Mild cognitive impairment
MMSE: Mini–Mental State Examination
MNI: Montreal Neurological Institute
NC: Normal control
PET: Positron emission tomography
pICA: Parallel independent component analysis
ROI: Regions of interest
rs-fMRI: Resting state functional connectivity MRI
SEN: Sensitivity
SN: Subcortical networks
SPE: Specificity
Tau PET: AV1451
VN: Visual network
