# Supplementary material for: Brain network alterations in individuals with and without mild cognitive impairment: parallel independent component analysis of AV1451 and AV45 positron emission tomography
Source: BMC Psychiatry. 2019 Jun 3;19:165. doi: 10.1186/s12888-019-2149-9 (PMC6547610; doi:10.1186/s12888-019-2149-9)
Supplement: Supplementary file 1 — Acquisition parameters of PET data. We have revised in the manuscript. (DOCX 13 kb) [file 12888_2019_2149_MOESM1_ESM.docx]

Supplementary Information

1. Acquisition parameters of PET data

1. **Image acquisition process:** In ADNI database, all PET Data were acquired using Siemens, GE and Philips PET scanners at resting-state. For the AV-1451 PET approximately 10 mCi of [18F]-AV-1451 was injected intravenously; after a 75-minute uptake, participants were imaged for 30 minutes using continuous list-mode data acquisition on a Siemens mCT and subsequently rebinned into six 5-minute frames. For AV-45 PET approximately 10 mCi of [18F]-AV-45 was injected intravenously; after a 50-minute uptake, participants were imaged for 20 minutes using continuous list-mode data acquisition on a Siemens mCT and subsequently rebinned into four 5-minute frames.
2. **Image uniform registration process:** Raw AV-1451 and AV-45 PET images were processed to remove the possible differences resulting from scanner differences. For a given subject, each frame was coregistered to the first frame, and then all frames were averaged to generate a single average image. The averaged image was reoriented and filtered into a standard 160×160×96 voxel image grid with 1.5 mm cubic voxels. This image grid is oriented such that the anterior-posterior axis of the subject is parallel to the AC-PC line. This is referred to as “AC-PC” space in the LONI search program. This standardized image then serves as a reference image for all PET scans on that subject. The individual frames from each PET scan (the baseline study as well as all subsequent studies (6-month scan, 12-month scan, etc.) are co-registered to this baseline reference image. By doing the co-registration from the original raw image data to a standardized space in a single step, only one interpolation of the image data is required, and thus resolution degradation by interpolation is kept to a minimum, and is the same for all scans. An averaged image is generated from the “AC-PC” co-registered frames and then intensity normalized using a subject-specific mask so that the average of voxels within the mask is exactly one. Both the spatial orientation (AC-PC) and the intensity normalization of the image are intended as a starting point for subsequent analyses. With a standardized image matrix, PET data from different scanner models can be compared more easily. So these images sets spatial re-orientation and intensity normalization of scans has occurred. The resulting images were smoothed with 8 mm FWHM Gaussian kernels. Through these methods, our data can be analyzed under a unified standard. Related changes have been marked in the paper.
